# Personal Health Libraries for People Returning From Incarceration: Protocol for a Qualitative Study

**DOI:** 10.2196/44748

**Published:** 2023-05-03

**Authors:** Marisol Foumakoye, Meredith Campbell Britton, Emile Ansari, Monya Saunders, Terika McCall, Emily A Wang, Lisa B Puglisi, T Elizabeth Workman, Qing Zeng-Treitler, Yin Ying, Shira Shavit, Cynthia A Brandt, Karen H Wang

**Affiliations:** 1 SEICHE Center for Health Justice Yale School of Medicine Yale University New Haven, CT United States; 2 Equity Research and Innovation Center Yale School of Medicine Yale University New Haven, CT United States; 3 Community Alliance for Research & Engagement Yale School of Public Health Yale University New Haven, CT United States; 4 Division of Health Informatics, Department of Biostatistics Yale School of Public Health Yale University New Haven, CT United States; 5 Section of Biomedical Informatics and Data Science Yale School of Medicine Yale University New Haven, CT United States; 6 Center for Interdisciplinary Research on AIDS Yale School of Public Health Yale University New Haven, CT United States; 7 Center for Biomedical Informatics George Washington University Washington, DC United States; 8 Department of Veterans Affairs VA Healthcare Systems West Haven, CT United States; 9 Transitions Clinic Network San Francisco, CA United States; 10 Department of Family and Community Medicine University of California San Francisco San Francisco, CA United States

**Keywords:** carceral settings, personal health information technology, qualitative research, systemic oppression, transition from incarceration

## Abstract

**Background:**

Individuals released from carceral facilities have high rates of hospitalization and death, especially in the weeks immediately after their return to community settings. During this transitional process, individuals leaving incarceration are expected to engage with multiple providers working in separate, complex systems, including health care clinics, social service agencies, community-based organizations, and probation and parole services. This navigation is often complicated by individuals’ physical and mental health, literacy and fluency, and socioeconomic status. Personal health information technology, which can help people access and organize their health information, could improve the transition from carceral systems to the community and mitigate health risks upon release. Yet, personal health information technologies have not been designed to meet the needs and preferences of this population nor tested for acceptability or use.

**Objective:**

The objective of our study is to develop a mobile app to create personal health libraries for individuals returning from incarceration to help bridge the transition from carceral settings to community living.

**Methods:**

Participants were recruited through Transitions Clinic Network clinic encounters and professional networking with justice-involved organizations. We used qualitative research methods to assess the facilitators and barriers to developing and using personal health information technology for individuals returning from incarceration. We conducted individual interviews with people just released from carceral facilities (n=~20) and providers (n=~10) from the local community and carceral facilities involved with the transition for returning community members. We used rigorous rapid qualitative analysis to generate thematic output characterizing the unique circumstances impacting the development and use of personal health information technology for individuals returning from incarceration and to identify content and features for the mobile app based on the preferences and needs of our participants.

**Results:**

As of February 2023, we have completed 27 qualitative interviews with individuals recently released from carceral systems (n=20) and stakeholders (n=7) who support justice-involved individuals from various organizations in the community.

**Conclusions:**

We anticipate that the study will characterize the experiences of people transitioning from prison and jails to community settings; describe the information, technology resources, and needs upon reentry to the community; and create potential pathways for fostering engagement with personal health information technology.

**International Registered Report Identifier (IRRID):**

DERR1-10.2196/44748

## Introduction

In a typical year, more than 10 million people cycle in and out of prisons and jails. Incarcerated individuals predominantly come from racial and ethnic minority groups and are of low-income status [1]. Those with a history of incarceration have chronic medical conditions—such as diabetes, hypertension, hepatitis C, depression, and substance use disorders—higher than the general population [2-6]. Upon release, these individuals bear high risks of death and hospitalization from preventable conditions [2,7-9]. Medicare beneficiaries recently released from correctional facilities have higher hospitalization rates and an increased risk of mortality compared with the general population [10,11]. A Washington state study found that individuals recently released from prison had a 2-fold increase in the risk of mortality in the year after release compared to the general population [7].

During incarceration, people reside in a highly controlled environment with constitutionally guaranteed access to health care services and little or no autonomy or opportunity to gain skills in self-management of medications, exercise, or diet [12,13]. This lack of autonomy may adversely affect an individual’s ability to manage their health upon release [12-14]. In total, 40% of individuals are diagnosed with a new chronic condition while incarcerated and do not have any experience managing that illness in a community setting [15]. Many individuals are released from carceral settings without any discharge planning and have no information on their health records, their medications, or their recent lab results [16]. Improving health outcomes in this population is further complicated given the immediacy of people’s social needs and legal obligations, including securing housing, food, and employment, as well as meeting the mandates of community supervision. After incarceration, people have an increased rate of homelessness, and incarceration leads to increased housing vulnerability due to loss of housing during incarceration, decreased eligibility for employment and public housing, and disrupted community ties [17]. In addition, more than 90% of formerly incarcerated persons meet the United States Department of Agriculture criteria for food insecurity [18]. Although many individuals have at least 1 chronic medical condition on release, 80% are released without health insurance or having a primary care provider [19,20]. Meeting these needs requires individuals just released from incarceration to engage with many different health, social, and legal entities to organize and manage their information.

Personal health information technologies are untapped resources that could facilitate improved access and organization of information, especially in the period after release. In addition, personal health information technologies increase patient engagement in health and self-efficacy efforts by organizing health information, improving communication with providers, and coordinating care. Research on personal health information technologies has mostly focused on patient portals (electronic personal health records tethered to institutional electronic health records), which has helped improve adherence to medications and quality measures for chronic conditions [21-25]. Yet, research has demonstrated this one-size-fits-all approach to designing personal health information technologies has resulted in low utilization of patient portals and barriers to use among racial and ethnic minority groups, people with low socioeconomic status, and people with limited health literacy [22,26,27]. In addition to low utilization and the challenges with use, patient portals do not have the desired functionality to aggregate information between different health systems and other organizations, like community-based social service organizations and carceral health facilities. Furthermore, the existing personal health information technologies that are not tied to an electronic health record aggregate information across multiple different sources but do not have features that address health literacy, concerns of privacy and security, and other barriers to technology use [28-30]. Existing personal health information technology, such as personal health libraries, has not been designed to meet the needs and preferences of people recently released from incarceration, which is critical for its acceptance and use [11,31]. Incorporating input from intended users and knowledge of their technology use and behaviors can help diminish potential intervention-generated inequalities [32,33]. The objective of this study is to explore the facilitators and barriers to using personal health information technology with formerly incarcerated persons and other community stakeholders. This study will inform the design, development, and usability testing of personal health libraries created for people with histories of incarceration to facilitate their transition from carceral to community settings.

## Methods

### Study Design, Study Setting, and Participants

We will use qualitative methods and conduct semistructured interviews with people just released from jails or prisons (anticipated n=20) and community and correctional stakeholders (anticipated n=10) to understand the individual, organization, and systemic factors that shape people’s access to health and technology supports after release from incarceration. The following study protocol was reviewed and approved by the Yale University Human Subjects Research Committee Institutional Review Board (200028862). The research will be conducted in partnership with a primary-care clinic that cares for people who have chronic health conditions and histories of incarceration. The clinic, located in the northeastern United States, is part of the Transitions Clinic Network (TCN), a national network of primary-care clinics that integrates community health workers (CHWs) who have lived experience of incarceration into clinical teams [3,15]. Additionally, the TCN clinic has collaborations with local organizations that support individuals released from correctional settings. We will create an advisory board for this project that includes clinicians and service providers who care for people with a history of incarceration, CHWs with a history of incarceration, and correctional system administrators. The advisory board will provide overarching feedback on study design, data collection, and data analysis.

#### Recruitment and Interviewing of Formerly Incarcerated Individuals

One team member (MS), who is a CHW in the clinic, will recruit potential participants through TCN clinic encounters and professional networking with justice-involved organizations. To be eligible for this interview, patients need to be at least 18 years old, carry a diagnosis of at least 1 chronic disease (eg, diabetes, heart disease, kidney disease, arthritis), be English-speaking, and have been released from jail or prison within the past year.

#### Recruitment and Interviewing of Stakeholders

Team members (MF, MCB, MS, KW) will use professional networks and snowball sampling to recruit stakeholder participants from social service agencies, community organizations, and carceral facilities. To be eligible for this portion, participants need to have direct experience working with people who have recently been released from incarceration.

All interested and eligible people will be given study-informed consent forms with detailed information about the purpose of the study, their rights as research participants, and contact information for further questions. A team member will schedule in-person interviews in physical or web-based spaces that are convenient for participants. All participants, both formerly incarcerated individuals and community stakeholders, will receive a US $50 gift card at the end of the interview in appreciation for their time.

### Data Collection

The team developed 2 interview guides using the constructs of the technology acceptance model and input from stakeholders who are part of the TCN professional network (see Multimedia Appendices 1 and 2 for interview guides) [34,35]. The interview guide for people just released from incarceration includes questions about participants’ experiences with technology, information-seeking behavior, and needs during their transition from incarceration to the community. Questions include:

- What experiences did you have with technology before incarceration? During incarceration?
- Describe some of the initial things you needed to figure out when you were released.
- How do you figure out what to do next?
- How do you organize your information?

Participants will also be asked to give feedback on wireframe models of a personal health library and share examples of information they would like to track or manage within a digital app, such as their identification card or address of residency prescription medications, or applications for education, employment, or financial assistance.

The stakeholder interview guide seeks to obtain information about technology access and information-seeking behaviors from a service provider perspective. Questions include:

- What kind of information do you collect and store on individuals who are in or just released from correctional settings?
- What type of information do you want to help you with your ability to promote health for the individuals you work with?
- What are your concerns about clients using technology to access their health information?

The team will iteratively refine both interview guides throughout data collection to incorporate emergent ideas into future interviews.

Three researchers will serve as interviewers for the study: 2 are experienced qualitative researchers (MF, MCB) while the third will be trained as part of the larger grant aims (MS). All interviews will be audio-recorded and professionally transcribed. Identifying information will be removed from the transcript to preserve participant confidentiality.

### Data Analysis

Four team members (MF, MCB, MS, KW), including the 3 interviewers, will serve on the analytic team. This structure will support a closeness with the data and a collaborative analytic process [36]. The team members’ experiences are professionally and personally diverse and span more than 10 years in the care of structurally marginalized populations, including expertise in justice research (MF), social work research (MCB), community health work and community advocacy (MS), medicine and community-engaged informatics (KW). They will use a rapid qualitative analysis to efficiently process and distribute data to the larger study team [37,38], so that the findings are incorporated into the user-centered design and software development process. The analytic team will develop a template to organize the salient findings from each individual interview [38]. The template contains sections describing each participant’s experiences with preparation for release from incarceration, perspectives on priorities for people after they are released from incarceration, and the type of information sought, shared, and used during the transition process from carceral settings to the community.

The analysts will complete summaries for each interview independently, with at least 2 analysts assigned to each transcript. They will then convene as a group to discuss their work and further explore the data through questions and comparisons with other study interviews, field experiences, and research findings. The team will record their progress, including ongoing thematic analysis through memoing and visualizations [39,40]. All written (transcripts, summaries, memos) and graphic (visualizations) data will be uploaded to Dedoose (version 9.0.17; Socio-cultural Research Consultants, LLC) for data storage, sharing, and retrieval. The summaries and visualizations will be sent to the larger research team to inform the ongoing design and development of a personal health library for individuals transitioning from carceral settings. The summary template includes a section that will be used to develop a qualitative codebook that can be applied to all transcripts for further analysis. This coding process will also facilitate the retrieval of aggregated, deidentified data for future study needs [41,42].

Following rapid qualitative analysis, the team will conduct interpretation sessions (led by TM) and develop an affinity diagram [43]. By creating an affinity diagram, the team can take the large amounts of data gathered during the interviews and organize them into groups or themes based on their relationships. The benefit of creating the affinity diagram is that it can reveal common issues and themes across all users and help to identify key requirements for developing the personal health library app. The purpose of the interpretation session is to turn the data gathered from the interviews into draft models for design. The study results will be disseminated to study participants and the advisory board for reflection and feedback.

### Ethics Approval

The institutional review board at Yale School of Medicine approved the study and all participants gave informed consent (2000028862).

## Results

As of February 2023, we have completed 27 interviews with people just released from carceral systems (n=20) and stakeholders (n=7). The data analysis derived from the interviews will inform the development of user requirements, identify facilitators and barriers to personal health library use with our population of interest, capture emergent needs and priorities during the transition process from correctional to community settings, and build a comprehensive codebook for further analysis. The interviews with people with a recent history of incarceration have elucidated a wide range of social and health services that they interact with upon release, such as identity verification and obtainment of identity documentation, 211 services (information and referral service organization in Connecticut servicing individuals with utility assistance, food, housing, child care, after school programs, elder care, and crisis intervention), the Department of Social Services that delivers and funds a wide range of programs and services as Connecticut's multifaceted health and human services agency, and the Department of Mental Health and Addiction Services, whose purpose is to promote and administer comprehensive, recovery-oriented services in the areas of mental health treatment and substance abuse prevention and treatment throughout Connecticut [44-46]. We are purposively sampling to recruit more female participants with recent histories of incarceration, as well as stakeholders affiliated with specific areas of need.

## Discussion

### Anticipated Findings

People recently released from carceral settings face a unique set of challenges when they return to the community [47]. They may face challenges with meeting their basic needs, such as being unhoused, unemployed, and food insecure, in addition to challenges maintaining their health and well-being when entering back into the community from prison or jail. In this transition back to the community, personal health libraries may be the bridge that facilitates access to information about community resources as well as facilitate the organization and preparation of personal health information, emergency contacts, community providers, and employment opportunities. From our qualitative analysis, we will have a rich understanding of the experiences of those previously imprisoned and their perspectives on the use of technology, their concern for security and safety use with technology, how they organize information that is important to them, and specifically how technology may or may not be leveraged for their personal health needs. In addition, by leveraging a participatory design process, the findings produced by this study will result in a nuanced understanding of the facilitators and barriers to the design, development, and utilization of personal health information technologies among people returning to the community from carceral settings. These findings from the interviews and the participatory design process may lead to increased personal health engagement through technology use.

### Conclusion

Millions of individuals leave jails and prisons each year, and in this transition, many experience poor health outcomes. Personal health information technologies, which are currently underutilized, may positively impact their health by improving individual self-efficacy and by facilitating communication between the health system and social service providers, and control of information by patients themselves. To improve transitions of care from carceral to the community health system, we need to have targeted and feasible solutions to capitalize on the benefits of personal health information technologies.

## Appendix

Multimedia Appendix 1 Interview guide for stakeholders.

Multimedia Appendix 2 Interview guide for formerly incarcerated persons.

Multimedia Appendix 3 Peer-review reports from the Biomedical Informatics, Library and Data Sciences Review Committee - National Library of Medicine (National Institutes of Health, USA).

## Abbreviations

CHW: community health worker
TCN-CT: Transitions Clinic Network, Connecticut

## Data Availability

The data sets generated during and analyzed during the current study are not publicly available due to ongoing data collection from participants but may be made available from the corresponding author on reasonable request.

## References

[1] Minton TD, Zeng Z (2016). Jail inmates in 2015. US Department of Justice: Bureau of Justice Statistics.

[2] Binswanger IA, Nowels C, Corsi KF, Long J, Booth RE, Kutner J, Steiner JF (2011). "From the prison door right to the sidewalk, everything went downhill," a qualitative study of the health experiences of recently released inmates. Int J Law Psychiatry.

[3] Shavit S, Aminawung JA, Birnbaum N, Greenberg S, Berthold T, Fishman A, Busch SH, Wang EA (2017). Transitions clinic network: challenges and lessons in primary care for people released from prison. Health Aff (Millwood).

[4] Wildeman C, Wang EA (2017). Mass incarceration, public health, and widening inequality in the USA. Lancet.

[5] Binswanger I A, Krueger P M, Steiner J F (2009). Prevalence of chronic medical conditions among jail and prison inmates in the USA compared with the general population. J Epidemiol Community Health.

[6] Wang EA, Redmond N, Dennison Himmelfarb CR, Pettit B, Stern M, Chen J, Shero S, Iturriaga E, Sorlie P, Diez Roux AV (2017). Cardiovascular disease in incarcerated populations. J Am Coll Cardiol.

[7] Binswanger IA, Stern MF, Deyo RA, Heagerty PJ, Cheadle A, Elmore JG, Koepsell TD (2007). Release from prison: a high risk of death for former inmates. N Engl J Med.

[8] Wang EA, Wang Y, Krumholz HM (2013). A high risk of hospitalization following release from correctional facilities in medicare beneficiaries: a retrospective matched cohort study, 2002 to 2010. JAMA Intern Med.

[9] Rich JD, Wakeman SE, Dickman SL (2011). Medicine and the epidemic of incarceration in the United States. N Engl J Med.

[10] Greenberg GA, Rosenheck RA (2008). Jail incarceration, homelessness, and mental health: a national study. Psychiatr Serv.

[11] Surkan PJ, Puglisi LB, Butler K, Elmi N, Zachary WW (2021). A roadmap for cardiovascular care after release from incarceration: uses of a smartphone application. J Am Med Inform Assoc.

[12] Thomas EH, Wang EA, Curry LA, Chen PG (2016). Patients' experiences managing cardiovascular disease and risk factors in prison. Health Justice.

[13] Hunter Buskey RN, Mathieson K, Leafman JS, Feinglos MN (2015). The effect of blood glucose self-monitoring among inmates with diabetes. J Correct Health Care.

[14] Eves A, Gesch B (2003). Food provision and the nutritional implications of food choices made by young adult males, in a young offenders' institution. J Hum Nutr Diet.

[15] Wang EA, Hong CS, Shavit S, Sanders R, Kessell E, Kushel MB (2012). Engaging individuals recently released from prison into primary care: a randomized trial. Am J Public Health.

[16] Prison Policy Initiative.

[17] Garcia-Grossman I, Kaplan L, Valle K, Guzman D, Williams B, Kushel M (2022). Factors associated with incarceration in older adults experiencing homelessness: results from the HOPE HOME study. J Gen Intern Med.

[18] Wang EA, Zhu GA, Evans L, Carroll-Scott A, Desai R, Fiellin LE (2013). A pilot study examining food insecurity and HIV risk behaviors among individuals recently released from prison. AIDS Educ Prev.

[19] Gates A, Artiga S, Rudowitz R (2014). Health coverage and care for the adult criminal justice-involved population. The Henry J Kaiser Family Foundation.

[20] Wang EA, White MC, Jamison R, Goldenson J, Estes M, Tulsky JP (2008). Discharge planning and continuity of health care: findings from the San Francisco county jail. Am J Public Health.

[21] Irizarry T, DeVito Dabbs A, Curran CR (2015). Patient portals and patient engagement: a state of the science review. J Med Internet Res.

[22] Sarkar U, Lyles CR, Parker MM, Allen J, Nguyen R, Moffet HH, Schillinger D, Karter AJ (2014). Use of the refill function through an online patient portal is associated with improved adherence to statins in an integrated health system. Med Care.

[23] Ralston JD, Hirsch IB, Hoath J, Mullen M, Cheadle A, Goldberg HI (2009). Web-based collaborative care for type 2 diabetes: a pilot randomized trial. Diabetes Care.

[24] Simon GE, Ralston JD, Savarino J, Pabiniak C, Wentzel C, Operskalski BH (2011). Randomized trial of depression follow-up care by online messaging. J Gen Intern Med.

[25] Tenforde M, Nowacki A, Jain A, Hickner J (2012). The association between personal health record use and diabetes quality measures. J Gen Intern Med.

[26] Sarkar U, Gourley GI, Lyles CR, Tieu L, Clarity C, Newmark L, Singh K, Bates DW (2016). Usability of commercially available mobile applications for diverse patients. J Gen Intern Med.

[27] Tieu L, Sarkar U, Schillinger D, Ralston JD, Ratanawongsa N, Pasick R, Lyles CR (2015). Barriers and facilitators to online portal use among patients and caregivers in a safety net health care system: a qualitative study. J Med Internet Res.

[28] Zapata BC, Hernández Niñirola A, Fernández-Alemán J, Toval A (2014). Assessing the privacy policies in mobile personal health records.

[29] Kharrazi H, Chisholm R, VanNasdale D, Thompson B (2012). Mobile personal health records: an evaluation of features and functionality. Int J Med Inform.

[30] Maloney FL, Wright A (2010). USB-based personal health records: an analysis of features and functionality. Int J Med Inform.

[31] Veinot TC, Mitchell H, Ancker JS (2018). Good intentions are not enough: how informatics interventions can worsen inequality. J Am Med Inform Assoc.

[32] McCall T, Ali MO, Yu F, Fontelo P, Khairat S (2021). Development of a mobile app to support self-management of anxiety and depression in African American women: usability study. JMIR Form Res.

[33] McCall T, Threats M, Pillai M, Lakdawala A, Bolton CS 3rd (2022). Recommendations for design of a mobile application to support management of anxiety and depression among Black American women. Front Digit Health.

[34] Venkatesh V, Morris MG, Davis GB, Davis FD (2003). User acceptance of information technology: toward a unified view. MIS Quarterly.

[35] Venkatesh V, Thong JYL, Xu X (2012). Consumer acceptance and use of information technology: extending the unified theory of acceptance and use of technology. MIS Quarterly.

[36] Saldaña J (2021). The Coding Manual for Qualitative Researchers, 4th ed.

[37] Vindrola-Padros C, Johnson GA (2020). Rapid techniques in qualitative research: a critical review of the literature. Qual Health Res.

[38] Hamilton A (2013). Qualitative methods in rapid turn-around health services research. Health Services Research & Development Cyberseminar.

[39] Glegg SMN (2019). Facilitating interviews in qualitative research with visual tools: a typology. Qual Health Res.

[40] Birks M, Chapman Y, Francis K (2008). Memoing in qualitative research: probing data and processes. J Res Nurs.

[41] MacQueen KM, McLellan E, Kay K, Milstein B (1998). Codebook development for team-based qualitative analysis. CAM Journal.

[42] Nowell LS, Norris JM, White DE, Moules NJ (2017). Thematic analysis: striving to meet the trustworthiness criteria. Int J Qual Methods.

[43] Holtzblatt K, Beyer H (2015). Design for life. Contextual Design.

[44] United Way of Connecticut.

[45] Department of Mental Health and Addiction Services. LinkedIn.

[46] Connecticut State Department of Social Services.

[47] (2001). From prison to home: the effect of incarceration and reentry on children, families, and communities. US Department of Health and Human Services.

