# Supplementary material for: Personal Health Libraries for People Returning From Incarceration: Protocol for a Qualitative Study
Source: JMIR Res Protoc. 2023 May 3;12:e44748. doi: 10.2196/44748 (PMC10193212; doi:10.2196/44748)
Supplement: Multimedia Appendix 3 [file resprot_v12i1e44748_app3.docx]

**PROGRAM CONTACT: ALAN VANBIERVLIET 301-594-1297**

[**alan.vanbiervliet@nih.gov**](mailto:alan.vanbiervliet@nih.gov)

**SUMMARY STATEMENT**

**( Privileged Communication )**

***Release Date:* 07/30/2020**

***Revised Date:***

**Principal Investigator WANG, KAREN H**

***Application Number:* 1 R01 LM013477-01**

**Applicant Organization: YALE UNIVERSITY**

***Review Group:* BLR**

**Biomedical Informatics, Library and Data Sciences Review Committee**

| ***Meeting Date:*** | **06/18/2020** | ***RFA/PA:* PAR19-072** |
| --- | --- | --- |
| ***Council:*** | **OCT 2020** | ***PCC:* PRPHLV** |
| ***Requested Start:*** | **12/01/2020** |  |

***Project Title:* Personal Health Libraries for Formerly Incarcerated Individuals**

***SRG Action:* Impact Score:29 Percentile:13 #**

***Next Steps:* Visit https://grants.nih.gov/grants/next_steps.htm Human Subjects: 48-At time of award, restrictions will apply**

**Animal Subjects: 10-No live vertebrate animals involved for competing appl.**

**Gender: Minority:**

**Age:**

**1A-Both genders, scientifically acceptable**

**1A-Minorities and non-minorities, scientifically acceptable 3A-No children included, scientifically acceptable**

| **Project**  **Year** | **Direct Costs**  **Requested** | **Estimated**  **Total Cost** |
| --- | --- | --- |
| **1** | **250,000** | **420,246** |
| **2** | **250,000** | **420,246** |
| **3** | **250,000** | **420,246** |
| **4** | **250,000** | **420,246** |

**TOTAL**

**1,000,000**

**1,680,985**

**ADMINISTRATIVE BUDGET NOTE: The budget shown is the requested budget and has not been adjusted to reflect any recommendations made by reviewers. If an award is planned, the costs will be calculated by Institute grants management staff based on the recommendations outlined below in the COMMITTEE BUDGET RECOMMENDATIONS section.**

**EARLY STAGE INVESTIGATOR NEW INVESTIGATOR**

## 1R01LM013477-01 Wang, Karen

**EARLY STAGE INVESTIGATOR NEW INVESTIGATOR**

**PROTECTION OF HUMAN SUBJECTS UNACCEPTABLE RESUME AND SUMMARY OF DISCUSSION:**

The principal investigator (PI) proposes to develop a personal health library (PHL) mobile application for formerly incarcerated individuals (FII) to help them to make the healthcare transition from prisons to communities. This is a highly significant project with a potential to reduce health inequity for this marginalized population that has been stigmatized and disproportionately impacted by various chronic conditions. The innovation is the application itself, to connect the segregated prison electronic health record (EHR) with the outside settings. Three aims are clearly defined, supported by preliminary data. They will use semantic indexing that will not require high health/technology literacy. The investigators have rich experience in health disparities research, community engagement, social determinants, and informatics. The environment is outstanding, with good connections to prisons, to prison-related health centers, and other stakeholders. However, the PHL seems to be limited to EHR (passively collecting/displaying data) instead of as a knowledge resource for active decision support of their health concerns. The use of the PHL appears to be at random without integrating into a flow. While the proposed scope is large and complex, the investigators lack practical connection to clinical or operational informatics research aspects of the proposed work. Despite the weaknesses, the enthusiasm is still high due to its potentially high impact of this disparate FII population.

## DESCRIPTION (provided by applicant):

Individuals released from correctional facilities have high rates of hospitalization and death, especially in the weeks following release. Disproportionately poor and of racial and ethnic minority groups, they are already a high-risk group for poor health outcomes. The transition back to the community is marked with additional difficulties navigating the healthcare, community correctional, and social service systems and structural barriers to obtaining healthcare, housing, and employment. Individuals released from correctional facilities must engage with many providers, which presents challenges organizing and acting on information received from various organizations. Personal health information technologies are untapped resources which could improve the transition from corrections to the community and mitigate health risks. There is an urgent need to develop personal health information technologies in partnership with formerly incarcerated individuals and test their efficacy in improving health outcomes. Using these technologies, formerly incarcerated individuals could organize, understand and act on various sources of information, leading to improved self-efficacy and improved health outcomes. Yet, there has not been a personal health information technology designed to meet the needs and preferences of this population, which is critical for its acceptance and use. The long-term goal is to improve the health of formerly incarcerated people, facilitated by their use of personal health information technologies. The overall objective of this proposal is to develop and test strategies, in partnership with formerly incarcerated people, to increase acceptance and use of a personal health library (PerHL) mobile app.

Preliminary data indicate that using a participatory health informatics approach to engage people with histories of incarceration can lead to acceptance and use of a health technology. The central hypothesis of our study is that developing and refining PerHL in partnership with formerly incarcerated people will increase its acceptance and use. To test this hypothesis, we propose three aims: (1) Assess the facilitators and barriers of the development and use of personal health information technology for formerly incarcerated individuals, (2) Develop and refine PerHL for formerly incarcerated individuals, and (3) Conduct a pilot randomized trial to test the acceptance and use of PerHL among formerly incarcerated individuals. We will leverage the expertise and infrastructure of Transitions Clinic Network,

a national network of primary care clinics for formerly incarcerated people, and a multidisciplinary team of informaticists, clinical providers, formerly incarcerated individuals, and criminal justice leaders, creating a best-case scenario for developing PerHL. This study represents a new and substantial departure from the status quo by incorporating a participatory health informatics approach and state of the art informatics tools into the development and refinement of a personal health information technology for people recently released from correctional facilities.

## PUBLIC HEALTH RELEVANCE:

Ten million individuals, who are disproportionately of racial/ethnic minority groups, low socioeconomic status, and have a high burden of chronic conditions are released from jails and prisons annually. At release, they are at heightened risk of poor health outcomes given the abrupt change in environment and structural barriers to healthcare, housing, and employment and the lack of access to their own health information and social resources. The proposed study will develop and refine a person health library mobile app in partnership with formerly incarcerated individuals and establish the evidence to test its efficacy to improve health outcomes.

## CRITIQUES:

The criterion scores provided below are given by individual reviewers assigned to this application and the critiques from reviewers are presented “as is”, without significant modification or editing by NLM staff. These individual critiques and criterion scores reflect the opinions of these assigned reviewers, which may or may not reflect the final Impact/Priority Score or final outcome/decision of the whole committee.

## CRITIQUE 1

Significance: 1

Investigator(s): 1

Innovation: 3

Approach: 4

Environment: 2

**Overall Impact:** This is a proposal to develop a personal health library mHealth application for previously incarcerated individuals. There are three aims – assess barriers and facilitators through interview; developing the system; and a proposed trial to compare use of the system to use of the patient portal. The significance is high, with substantial health concerns, care fragmentation, and high burden on these individuals to manage their care at transitions. The investigator mix appears uniquely able to engage and address the needs of this underserved population, yet they lack practical connection to clinical or operational informatics (despite the inclusion of Dr. Brandt) and have limited information about the developer; the last is important because the proposed scope is large and complex. The innovation of the application itself – a PHL to connect the segregated world of the prison health care records to other health care settings – would be strong, especially in their assessment of facilitators and usability, as would some of the specific mentioned techniques, such as OCR of paper forms by patients themselves, but there is a lack of specificity on how these would be applied that reduces innovative potential. The approach has strong qualitative steps and is likely to gather clear needs and requirements to build a system, but is highly ambitious in its integrated picture, voice, video, unstructured and external structure data feeds. They propose to limit the specific concept domains extracted and perform usability testing, which somewhat mitigates this concern. The trial is interesting with outcomes focused on any use and perceptions of use, although the comparator isn’t well justified

or introduced (use of PHR), limiting the ability to understand the value added. The environment is strong, with good connections to prisons, to prison-related health centers, and other stakeholders.

## Significance:

**Strengths**

- - This vulnerable population – previously incarcerated individuals – have a large burden of illness and psychosocial needs, but the transition from jail to community has significant data gaps.
  - Understanding what will work for this population is both important and requires – as the proposal states – careful engagement of individuals as they have need of personal health libraries.

## Weaknesses

- - The lack of any connection to extant health information is concerning; the significance is lessened by the fact all information will be extracted de novo.

## Investigator(s):

**Strengths**

- - The principal investigator has an excellent breadth of experience in disparities research, community engagement, social determinants, and informatics.
  - Dr. Emily Wang (co-I) is a leading researcher at the Health Justice Lab.

## Weaknesses

- - Development, usability, and testing of application experience for novice users is limited.

## Innovation:

**Strengths**

- - Specific personal health libraries for recently incarcerated individuals do not exist, and innovative methods are being used here to make it possible for these individuals to self-create and use these.

## Weaknesses

- - The use of the technologies involved appear a bit at random and aren’t integrated into a flow that would truly innovate the way these individuals manage their care.

## Approach:

**Strengths**

- - The participatory-based research is strong.
  - The qualitative work to understand needs is clear and is likely to produce useful information about needs and high-level requirements.
  - The final study is interesting and has good outcomes and potential to add knowledge to the work.

## Weaknesses

- - The connection to any extant exchange standards is tenuous; although FHIR is mentioned, there is almost nothing about the way in which the concepts extracted will be connected to FHIR and potentially exchanged.
  - The lack of discussion of how the tool could be connected to EHR-based FHIR APIs is concerning; although CHCs are resource constrained, the frequency of EHRs in them is high.
  - The final study is comparing to a PHR, but it isn’t clear what functionalities exist in the EHR and how they will be compared. Thus, many of the measurements may not be comparing similar use-cases.

## Environment:

**Strengths**

- - The major strengths of the environment are the connection to the population as they leave jail and seek health care.

## Weaknesses

- - The connection to an operational data store / FHIR sandbox would be extremely helpful to see value early.

**Protections for Human Subjects:** Unacceptable Risks and/or Inadequate Protections

- - Highly vulnerable population. The risks are not clearly defined, which are loss of confidentiality and privacy; given their status as those previously incarcerated, these risks are incredibly important to weigh against. The tool itself will store PHI, and, although minimal risk, they need to highlight these.

Data and Safety Monitoring Plan: Acceptable

## Inclusion Plans:

- - Sex/Gender: Distribution justified scientifically
  - Race/Ethnicity: Distribution justified scientifically
  - For NIH-Defined Phase III trials, Plans for valid design and analysis:
  - Inclusion/Exclusion Based on Age: Distribution justified scientifically

**Vertebrate Animals:** Not Applicable **Biohazards:** Not Applicable **Resubmission:** Not Applicable **Renewal:** Not Applicable

**Revision:** Not Applicable

**Applications from Foreign Organizations:** Not Applicable

**Select Agents:** Not Applicable

**Resource Sharing Plans:** Unacceptable - There are very limited details on what will be shared.

## Authentication of Key Biological and/or Chemical Resources: Not Applicable

**Budget and Period of Support:** Recommend as Requested

## CRITIQUE 2

Significance: 3

Investigator(s): 2

Innovation: 3

Approach: 3

Environment: 1

**Overall Impact:** The investigators propose to develop a system that addresses an important set of patient information needs for a particularly vulnerable and underserved population – formerly incarcerated persons. The investigators have solid credentials for systems development using participatory design, although there is some concern that the PI lacks experience in the informatics research aspects of the proposed work. The resources available for the project appear to be excellent. The lack of inclusion of knowledge resources means that the patient library is really a personal health

record, albeit one that involves a good set of data sources. Enthusiasm is dampened somewhat by the lack of attention to the use of knowledge resources (which is surprising given some of the investigators’ experience with them) and by the appearance that the tool will be passive only – collecting and displaying data, but not providing active decision support or communicating with other systems to help address the user’s health problems.

## Significance:

**Strengths**

- - Formerly incarcerated individuals (FII) have special health needs, are underserved and are particularly vulnerable.
  - Information sources related to individuals’ health data are varied and important.

## Weaknesses

- - The lack of clear plans for addressing information needs other than those addressed by the personal health record detracts from the significance of the proposed project.
  - The significance of the proposed work is limited to FII who come from a correctional facility with an accessible electronic health record (EHR).

## Investigator(s):

**Strengths**

- - This is overall a strong team with vast collective experience.

## Weaknesses

- - The PI has some training in medical informatics but a bare minimum of scientific publications.

## Innovation:

**Strengths**

- - It is working with a unique target population.
  - The work will link a number of unique resources, including a correctional system EHR.

## Weaknesses

- - It seems to be missing an opportunity to understand the information needs of FIIs that go beyond patient data (i.e., knowledge resources).

## Approach:

**Strengths**

- - There is an excellent sequential participatory design process.
  - The platform design appears sound.
  - Access to the Transitions Clinic Network is an excellent complement to the EHR.
  - There is a large pilot trial involving 100 FFIs.
  - There are multiple relevant data sources, with a reasonable approach to consolidation.

## Weaknesses

- - In this proposal, “personal health libraries” seems to be limited to EHR data. Knowledge as a resource is mentioned once, briefly, in the Significance, with no details.
  - The predicament described at the bottom of page 67 seems to be a likely situation for an FFI. Other than providing access to his personal health information, it is not clear how the envisioned tool will help with other tasks (such as coordinating appointments).

## Environment:

**Strengths**

- - Access to appropriate research subjects and their data from multiple sources is a strong component of the proposed work.

## Weaknesses

- - None noted by the reviewer

**Protections for Human Subjects:** Acceptable Risks and/or Adequate Protections - Well-thought-out human subjects’ protocol

Data and Safety Monitoring Plan: Not Applicable

## Inclusion Plans:

- - Sex/Gender: Distribution justified scientifically
  - Race/Ethnicity: Distribution justified scientifically
  - For NIH-Defined Phase III trials, Plans for valid design and analysis: Not applicable
  - Inclusion/Exclusion Based on Age: Distribution justified scientifically
  - Study population will reflect demographics of FIIs

**Vertebrate Animals:** Not Applicable **Biohazards:** Not Applicable **Resubmission:** Not Applicable **Renewal:** Not Applicable

**Revision:** Not Applicable

**Applications from Foreign Organizations:** Not Applicable

**Select Agents:** Not Applicable

**Resource Sharing Plans:** Acceptable

## Authentication of Key Biological and/or Chemical Resources: Not Applicable

**Budget and Period of Support:** Recommend as Requested

## CRITIQUE 3

Significance: 2

Investigator(s): 1

Innovation: 3

Approach: 4

Environment: 1

**Overall Impact:** This R01 application seeks to address the health disparities and poor health outcomes of formerly incarcerated individuals by providing health information access via health technology. This marginalized population has been stigmatized and disproportionately impacted by various complex chronic conditions and social determinants of health. Community-based participatory research may have the potential to make significant advances in health equity. This project has the potential for high significance.

## Significance:

**Strengths**

- - This is a novel approach to promotion of health equity among formerly incarcerated individuals via health technology. This specific population has a high burden or chronic conditions and poor health outcomes, as well as, various challenges in re-integrating into society.
  - Personal health information technologies are an untapped resource that has not been previously utilized and there is an urgent need to improve this population’s health outcome.

## Weaknesses

- - None noted by the reviewer

## Investigator(s):

**Strengths**

- - The PI and investigators have a strong record of academic accomplishments and research experience to support the project.
  - The research team has an extensive publication record.
  - Letters of support are included.
  - The research team has established partnerships with various agencies to successfully conduct this project.

## Weaknesses

- - None noted by the reviewer

## Innovation:

**Strengths**

- - The research team proposes an innovative means to address health disparities in recently released incarcerated individuals by forming a partnership and engaging these individuals in actively participating in improving their health outcomes.
  - Stakeholders and end-users will be engaged in the development of a patient-centered workflow model with various health-related activities.
  - The application will use optical character recognition, speech to text, natural language techniques and build on semantic indexing that will not require high health or technology literacy.

## Weaknesses

- - No major weaknesses

## Approach:

**Strengths**

- - Preliminary studies have already been conducted.
  - There is a strong interdisciplinary team with the expertise in community-engaged health informatics.
  - Aims 1, 2 and 3 are clearly defined.
  - There are detailed descriptions and figures to support the research process.

## Weaknesses

- - Health information resources and specific information needs of formerly incarcerated individuals are not discussed.

## Environment:

**Strengths**

- - There are strong core facilities and infrastructure to support the research.

## Weaknesses

- - None

**Protections for Human Subjects:** Acceptable Risks and/or Adequate Protections Data and Safety Monitoring Plan: Acceptable

## Inclusion Plans:

- - Sex/Gender: Distribution justified scientifically
  - Race/Ethnicity: Distribution justified scientifically
  - For NIH-Defined Phase III trials, Plans for valid design and analysis:
  - Inclusion/Exclusion Based on Age: Distribution justified scientifically

**Vertebrate Animals:** Not Applicable **Biohazards:** Acceptable **Resubmission:** Not Applicable **Renewal:** Not Applicable

**Revision:** Not Applicable

**Applications from Foreign Organizations:** Not Applicable

**Select Agents:** Not Applicable

**Resource Sharing Plans:** Acceptable

## Authentication of Key Biological and/or Chemical Resources: Not Applicable

**Budget and Period of Support:** Recommend as Requested

## THE FOLLOWING SECTIONS WERE PREPARED BY THE SCIENTIFIC REVIEW OFFICER TO SUMMARIZE THE OUTCOME OF DISCUSSIONS OF THE REVIEW COMMITTEE, OR REVIEWERS’ WRITTEN CRITIQUES, ON THE FOLLOWING ISSUES:

**PROTECTION OF HUMAN SUBJECTS: UNACCEPTABLE –** This is a highly vulnerable population. The risks are not clearly defined.

## INCLUSION OF WOMEN PLAN: ACCEPTABLE INCLUSION OF MINORITIES PLAN: ACCEPTABLE INCLUSION ACROSS THE LIFESPAN: ACCEPTABLE VERTEBRATE ANIMAL: NOT APPLICABLE BIOHAZARDS: NOT APPLICABLE

**APPLICATIONS FROM FOREIGN ORGANIZATIONS: NOT APPLICABLE SELECT AGENTS: NOT APPLICABLE**

**RESOURCE SHARING PLANS: UNACCEPTABLE -** There are very limited details on what will be shared.

## AUTHENTICATION OF KEY BIOLOGICAL AND/OR CHEMICAL RESOURCES: NOT APPLICABLE

**COMMITTEE BUDGET RECOMMENDATIONS: The budget was recommended as requested.**

1 R01 LM013477-01 10 BLR WANG, K

# Footnotes for 1 R01 LM013477-01; PI Name: Wang, Karen H

# Ad hoc or special section application percentiled against "Total CSR" base.

NIH has modified its policy regarding the receipt of resubmissions (amended applications).See Guide Notice NOT-OD-18-197 at https://grants.nih.gov/grants/guide/notice-files/NOT-OD-18- 197.html. The impact/priority score is calculated after discussion of an application by averaging the overall scores (1-9) given by all voting reviewers on the committee and multiplying by 10. The criterion scores are submitted prior to the meeting by the individual reviewers assigned to an application, and are not discussed specifically at the review meeting or calculated into the overall impact score. Some applications also receive a percentile ranking. For details on the review process, see [http://grants.nih.gov/grants/peer_review_process.htm#scoring.](http://grants.nih.gov/grants/peer_review_process.htm#scoring)

MEETING ROSTER

Biomedical Informatics, Library and Data Sciences Review Committee NATIONAL LIBRARY OF MEDICINE

BLR 06/18/2020

Notice of NIH Policy to All Applicants: Meeting rosters are provided for information purposes only. Applicant investigators and institutional officials must not communicate directly with study section members about an application before or after the review. Failure to observe this policy will create a serious breach of integrity in the peer review process, and may lead to actions outlined in NOT-OD-14-073 at https://grants.nih.gov/grants/guide/notice-files/NOT-OD-14-073.html and NOT-OD-15-106 at

https://grants.nih.gov/grants/guide/notice-files/NOT-OD-15-106.html, including removal of the application from immediate review.

CHAIRPERSON(S)

HOLMES, JOHN H, PHD

PROFESSOR OF MEDICAL INFORMATICS IN EPIDEMIOLOGY DEPARTMENT OF BIOSTATISTICS, EPIDEMIOLOGY, AND INFORMATICS, PERELMAN SCHOOL OF MEDICINE AT

THE UNIVERSITY OF PENNSYLVANIA

CLARK, TIMOTHY W, PHD * ASSOCIATE PROFESSOR

PUBLIC HEALTH SCIENCES, DATA SCIENCE, AND NEUROLOGY

SCHOOL OF MEDICINE & SCHOOL OF DATA SCIENCE UNIVERSITY OF VIRGINIA

PHILADELPHIA, PA 19104 CHARLOTTESVILLE, VA 22903

MEMBERS

BENOS, PANAGIOLIS V, PHD * PROFESSOR AND VICE CHAIR

DEPARTMENT OF COMPUTATIONAL AND SYSTEMS BIOLOGY

UNIVERSITY OF PITTSBURGH PITTSBURGH, PA 15260

BURNS, GULLY A, DPHIL RESEARCH SCIENTIST

META, CHAN ZUCKERBERG INITIATIVE REDWOOD CITY, CA 94062

DAVULURI, RAMANA V, PHD PROFESSOR

DEPARTMENT OF PREVENTIVE MEDICINE

DIVISION OF HEALTH AND BIOMEDICAL INFORMATICS FEINBERG SCHOOL OF MEDICINE

NORTHWESTERN UNIVERSITY CHICAGO, IL 60611

DONALDSON, DEVAN R, PHD ASSISTANT PROFESSOR

DEPARTMENT OF INFORMATION AND LIBRARY SCIENCE LUDDY SCHOOL OF INFORMATICS, COMPUTING AND ENGINEERING

INDIANA UNIVERSITY

CALLAHAN, ALISON, PHD BLOOMINGTON, IN 47408

RESEARCH SCIENTIST

CENTER FOR BIOMEDICAL INFORMATICS RESEARCH STANFORD UNIVERSITY

STANFORD, CA 94305

CHEN, ELIZABETH S, PHD

INTERIM DIRECTOR OF THE CENTER FOR BIOMEDICAL INFORMATICS

ASSOCIATE PROFESSOR OF HEALTH SERVICES POLICY AND PRACTICE

ASSOCIATE DIRECTOR OF THE CENTER FOR BIOMEDICAL INFORMATICS, BROWN UNIVERSITY PROVIDENCE, RI 02912

CIMINO, JAMES J, MD DIRECTOR INFORMATICS INSTITUTE SCHOOL OF MEDICINE

UNIVERSITY OF ALABAMA AT BIRMINGHAM BIRMINGHAM, AL 35233

DORR, DAVID A, MD PROFESSOR AND VICE CHAIR

DEPARTMENT OF MEDICAL INFORMATICS AND CLINICAL EPIDEMIOLOGY

OREGON HEALTH & SCIENCE UNIVERSITY PORTLAND, OR 97239

DOUGLAS-WILLIAMS, TARA, MSLS LIBRARY MANAGER INFORMATION SERVICES

MOREHOUSE SCHOOL OF MEDICINE ATLANTA, GA 30310

ELHADAD, NOEMIE, PHD ASSOCIATE PROFESSOR

DEPARTMENT OF BIOMEDICAL INFORMATICS COLUMBIA UNIVERSITY

NEW YORK, NY 10032

GRANNIS, SHAUN J, MD PATEL, CHIRAG J, PHD

REGENSTRIEF ENDOWED CHAIR AND PROFESSOR OF MEDICAL INFORMATICS

REGENSTRIEF VICE PRESIDENT FOR DATA AND ANALYTICS PROFESSOR OF FAMILY MEDICINE

INDIANA UNIVERSITY SCHOOL OF MEDICINE INDIANAPOLIS, IN 46203

HSU, WILLIAM, PHD * ASSOCIATE PROFESSOR

DEPARTMENT OF RADIOLOGICAL SCIENCES, BIOINFORMATICS, AND BIOENGINEERING UNIVERSITY OF CALIFORNIA, LOS ANGELES LOS ANGELES, CA 90024

KIEFE, CATARINA I, PHD, MD * CHAIR AND PROFESSOR

DEPARTMENT OF QUANTITATIVE HEALTH SCIENCES UNIVERSITY OF MASSACHUSETTS MEDICAL SCHOOL WORCESTER, MA 01605

ASSOICATE PROFESSOR

DEPARTMENT OF BIOMEDICAL INFORMATICS HARVARD MEDICAL SCHOOL

BOSTON, MA 02115

SMALHEISER, NEIL R, MD, PHD PROFESSOR

DEPARTMENT OF PSYCHIATRY COLLEGE OF MEDICINE

UNIVERSITY OF ILLINOIS AT CHICAGO CHICAGO, IL 60612

SWAMIDASS, S JOSHUA, MD, PHD ASSOCIATE PROFESSOR

DIVISION OF LABORATORY & GENOMIC MEDICINE FACULTY LEAD OF TRANSLATIONAL BIOINFORMATICS INSTITUTE FOR BIOINFORMATICS, DEPARTMENT OF IMMUNOLOGY & PATHOLOGY, WASHINGTON UNIVERSITY ST. LOUIS, MO 63110

KLEINBERG, SAMANTHA, PHD ASSOCIATE PROFESSOR COMPUTER SCIENCE

STEVENS INSTITUTE OF TECHNOLOGY HOBOKEN, NJ 07030

LEROY, GONDY, PHD *

ELLER FELLOW AND PROFESSOR MANAGEMENT INFORMATION SYSTEMS ELLER COLLEGE OF MANAGEMENT UNIVERSITY OF ARIZONA

TUCSON, AZ 85721

LICHTARGE, OLIVIER, MD, PHD

CULLEN ENDOWED CHAIR HOLDER AND PROFESSOR OF GENETICS

DEPARTMENT OF MOLECULAR AND HUMAN GENETICS BAYLOR COLLEGE OF MEDICINE

HOUSTON, TX 77030

LIN, JOSHUA K, MD *

ASSISTANT PROFESSOR, HARVARD MEDICAL SCHOOL DIVISION OF PHARMACOEPIDEMIOLOGY AND PHARMACOECONOMICS

BRIGHAM AND WOMEN'S HOSPITAL BOSTON, MA 02120

MADHAVAN, SUBHA, PHD ASSOCIATE PROFESSOR

INNOVATION CENTER FOR BIOMEDICAL INFORMATICS GEORGETOWN UNIVERSITY MEDICAL CENTER WASHINGTON, DC 20007

PAGE, JR., C DAVID, PHD PROFESSOR AND CHAIR

DEPARTMENT OF BIOSTATISTICS AND BIOINFORMATICS SCHOOL OF MEDICINE

DUKE UNIVERSITY DURHAM, NC 27710

TAYLOR, CASEY OVERBY, PHD ASSISTANT PROFESSOR

DEPARTMENT OF MEDICINE & BIOMEDICAL ENGINEERING SCHOOL OF MEDICINE

JOHNS HOPKINS UNIVERSITY BALTIMORE, MD 21205

SCIENTIFIC REVIEW OFFICER

HUANG, ZOE E, MD

CHIEF SCIENTIFIC REVIEW OFFICER DIVISION OF EXTRAMURAL PROGRAMS NATIONAL LIBRARY OF MEDICINE NATIONAL INSTITUTES OF HEALTH BETHESDA, MD 20892-7968

EXTRAMURAL SUPPORT ASSISTANT

HOLMES, LATANYA

EXTRAMURAL REVIEW ASSISTANT

NATIONAL INSTITUTE ON DEAFNESS & OTHER COMMUNICATIONS

BETHESDA, MD 20892

PROGRAM REPRESENTATIVE

SIM, HUA-CHUAN, MD CHIEF PROGRAM OFFICER

DIVISION OF EXTRAMURAL PROGRAMS NATIONAL LIBRARY OF MEDICINE NATIONAL INSTITUTES OF HEALTH BETHESDA, MD 20892

VANBIERVLIET, ALAN, PHD PROGRAM OFFICER

DIVISION OF EXTRAMURAL PROGRAMS NATIONAL LIBRARY OF MEDICINE NATIONAL INSTITUTES OF HEALTH BETHESDA, MD 20892

YE, JANE, PHD PROGRAM OFFICER

DIVISION OF EXTRAMURAL PROGRAMS NATIONAL LIBRARY OF MEDICINE NATIONAL INSTITUTES OF HEALTH BETHESDA, MD 20892

OTHER REVIEW STAFF

NICOLAS, MINDY, BA

REVIEW PROGRAM SPECIALIST DIVISION OF EXTRAMURAL PROGRAMS NATIONAL LIBRARY OF MEDICINE NATIONAL INSTITUTES OF HEALTH BETHESDA, MD 20892

OTHER

BARLETT, VALERIE PROGRAM SPECIALIST

DIVISION OF EXTRAMURAL PROGRAMS NATIONAL LIBRARY OF MEDICINE (DHHS/NIH) BETHESDA, MD 20892

FLORANCE, VALERIE, PHD

DIRECTOR, NLM - EXTRAMURAL PROGRAMS DIVISION OF EXTRAMURAL PROGRAMS NATIONAL LIBRARY OF MEDICINE

NATIONAL INSTITUTES OF HEALTH BETHESDA, MD 20892-7968

* Temporary Member. For grant applications, temporary members may participate in the entire meeting or may review only selected applications as needed.

Consultants are required to absent themselves from the room during the review of any application if their presence would constitute or appear to constitute a conflict of interest.
