# Supplementary material for: Personal Health Libraries for People Returning From Incarceration: Protocol for a Qualitative Study
Source: JMIR Res Protoc. 2023 May 3;12:e44748. doi: 10.2196/44748 (PMC10193212; doi:10.2196/44748)
Supplement: Supplementary file 2 [file resprot_v12i1e44748_app1.docx]

Appendix B

Stakeholder Interview Guide

1. Please describe your role and how long you've been in your position.

2. Please describe how you prepare people for their release from the correctional setting / work with people upon their release from the correctional setting.

   a. What steps were involved with the preparation?
   b. What resources/services do you arrange or provide? Such as social services, like place to live?
   c. How did you access information about the person? Databases? Use of technology? The person's social network?
   d. How much of this information was readily available?
   e. In a perfect world, what would be available to people upon release from jail or prison?

3. What kind of information do you collect and store on individuals in the correctional setting or under your supervision?

   a. What formats do you use to store this information?
   b. How do you store this information?
   c. How, if at all, do you share this information?
   d. Who, if at all, do you share this information with?

4. How is this information used? What types of information/data do you want to help you with your ability to provide healthcare or promote health for these individuals?

   a. What are issues you've run into getting this information?
   b. What are issues you've run into using this information?
   c. What makes things easier to obtain or use?
   d. How do you keep things organized? Examples: write down, calendar, phone notes etc.
   e. What have been the efforts to share information across the correctional and community health organizations?

5. What have been efforts to share individual level health information to those being released?

   a. Where is this information stored?
   b. Who currently has access to this information? (Examples: primary care doctor, nurse, social worker, case manager, community health worker)
   c. Is there a process for people to request their protected health information?
   d. What kind of resources (people, technology, policies) are needed to share data?
   e. What are technologies available to you that could share this information?
   f. What are polices that are likely need that facilitate this sharing directly with individuals?

6. What concerns do you have about shared data with individuals who are leaving or just left the correctional setting?

   a. Share your thoughts on individuals accessing their private health information.
   b. Is there information that you feel should not be shared? Why or why not?
