# Supplementary material for: Personal Health Libraries for People Returning From Incarceration: Protocol for a Qualitative Study
Source: JMIR Res Protoc. 2023 May 3;12:e44748. doi: 10.2196/44748 (PMC10193212; doi:10.2196/44748)
Supplement: Supplementary file 3 [file resprot_v12i1e44748_app2.docx]

Appendix A

Formerly Incarcerated Persons Interview Guide

1. What is your experience with incarceration?
    a. How long were you incarcerated?
    b. What were your experiences with tech/app/use inside (e.g., use of social media, access to computers)?
    c. Were you diagnosed with anything new related to mental health or physical health while incarcerated?
    d. Besides your medical records, are there any other records you'd like about yourself (kept by the system) that you'd like to take home with you? (School, dental, ID's, legal etc.)

2. Please describe how you prepared for your release from correctional settings.
    a. What resources/services did you need to arrange? Such as social services, like a place to live?
    b. What steps were involved with preparing for the services that you need?
    c. How did you get access to this information? Use of technology? Your social network?
    d. How much of this information was available to you from your community or peers inside?
    e. In a perfect world, what would be available to you upon your release from jail or prison?

3. Upon your release from corrections settings, describe some of the initial things you needed to figure out?

4. What are the issues you've run into getting this information? Using this information? What makes things easier to obtain or use?

5. What experiences did you have with technology before being incarcerated? During? After?
    a. What experiences have you used technology for health purposes? Share your thoughts on whether you'd want to access your mental or physical health records on a phone/app etc.
        a. What would you concern you if you were to lose your phone?

6. Let's talk about your private health information. Who has access to this information?
    a. Do you know how your private health information is shared? With whom?
    b. Where would you want your private health information stored?
    c. Who would you want to have access to it? Ex. Primary Care doctor, nurses, social worker?

7. We are developing an app to organize health information and non-health information. Let's look back at what you reported on what you needed upon release, what other information would you like access to on this app that would be helpful for you coming home?

8. Let's look at some of the information you have brought here today, describe it to me. Why are these examples important to you?
